# Characteristics of young lung cancer: Analysis of Taiwan's nationwide lung cancer registry focusing on epidermal growth factor receptor mutation and smoking status

**DOI:** 10.18632/oncotarget.9338

**Published:** 2016-05-13

**Authors:** Chia-Hung Hsu, Chien-Hua Tseng, Chun-Ju Chiang, Kuo-Hsuan Hsu, Jeng-Sen Tseng, Kun-Chieh Chen, Chih-Liang Wang, Chih-Yi Chen, Sang-Hue Yen, Chao-Hua Chiu, Ming-Shyan Huang, Chong-Jen Yu, Ying-Huang Tsai, Jin-Shing Chen, Chun-Ming Tsai, Teh-Ying Chou, Kuei-Chih Lin, Ming-Hsun Tsai, Wen-Chung Lee, Hsiu-Ying Ku, Tsang-Wu Liu, Tsung-Ying Yang, Gee-Chen Chang

**Affiliations:** ^1^ Division of Chest Medicine, Department of Internal Medicine, Taichung Veterans General Hospital, Taichung, Taiwan; ^2^ Division of Critical Care Medicine, Taichung Veterans General Hospital, Taichung, Taiwan; ^3^ Graduate Institute of Epidemiology and Preventive Medicine, College of Public Health, National Taiwan University, Taipei, Taiwan; ^4^ Taiwan Cancer Registry, Taipei, Taiwan; ^5^ Division of Critical Care and Respiratory Therapy, Department of Internal Medicine, Taichung Veterans General Hospital, Taichung, Taiwan; ^6^ Institute of Biomedical Sciences, National Chung Hsing University, Taichung, Taiwan; ^7^ Faculty of Medicine, School of Medicine, National Yang-Ming University, Taipei, Taiwan; ^8^ Department of Thoracic Medicine, Chang Gung Memorial Hospital, Taoyuan, Taiwan; ^9^ College of Medicine, Chang Gung University, Taoyuan, Taiwan; ^10^ Department of Surgery, Chung-Shang Medical University Hospital, Taichung, Taiwan; ^11^ Institute of Medicine, Chung Shan Medical University, Taichung, Taiwan; ^12^ Department of Oncology, Taipei Veterans General Hospital, Taipei, Taiwan; ^13^ Institute of Clinical Medicine, National Yang-Ming University, Taipei, Taiwan; ^14^ Division of Thoracic Oncology, Department of Chest Medicine, Taipei Veterans General Hospital, Taipei, Taiwan; ^15^ Department of Internal Medicine, Kaohsiung Medical University Hospital, Kaohsiung, Taiwan; ^16^ School of Medicine, Kaohsiung Medical University, Kaohsiung, Taiwan; ^17^ Department of Internal Medicine, National Taiwan University Hospital, Taipei, Taiwan; ^18^ College of Medicine, National Taiwan University, Taipei, Taiwan; ^19^ Department of Pulmonary and Critical Care Medicine, Chang Gung Memorial Hospital, Chiayi, Puzi City, Taiwan; ^20^ Department of Respiratory Therapy, Chang Gung University, Taoyuan, Taiwan; ^21^ Graduate Taipei Medicine College, Taipei, Taiwan; ^22^ Division of Thoracic Surgery, Department of Surgery, National Taiwan University Hospital, Taipei, Taiwan; ^23^ Department of Traumatology, National Taiwan University Hospital, Taipei, Taiwan; ^24^ Division of Thoracic Oncology, Department of Chest Medicine, Taipei Veterans General Hospital, Taipei, Taiwan; ^25^ Department of Pathology and Laboratory Medicine, Taipei Veterans General Hospital, Taipei, Taiwan; ^26^ National Institute of Cancer Research, National Health Research Institutes, Miaoli, Taiwan

**Keywords:** lung cancer, adenocarcinoma, young patients, EGFR

## Abstract

Lung cancer is relatively rare in young patients as the median age at diagnosis is 65–70 years. The main objective of this nationwide study was to investigate the characteristics of young lung cancer in Taiwan, especially the relationships among smoking behavior, *epidermal growth factor receptor* (*EGFR*) mutation, and age. The National Taiwan Lung Cancer Registry, a database contain detailed cancer statistics, was analyzed in this study for the period 2011–2012. Young lung cancer was defined as age ≦ 45 years. There were 21,536 lung cancer patients (13,187 men and 8349 women). Among these patients, 1074 (5.0%) were in the younger group, and 20,462 patients (95.0%) were in the older group. Female gender (48.8% versus 38.2%, *P* < 0.001), never-smokers (47.3% versus 43.8%, *P* = 0.015), and adenocarcinoma (70.4% versus 58.1%, *P* < 0.001) were more frequent in the younger group. While the *EGFR* mutation rate was lower in the younger group (52.5% versus 60.6%, *P* = 0.001), the primary site of lung cancer and stage distribution were not significantly different. If only adenocarcinoma patients were included in the analysis, female gender, older age, and never-smokers were more likely to have *EGFR* mutation. In conclusion, lung cancer in young patients (≦ 45 year-old) was associated with unique characteristics, with greater percentages of female patients, adenocarcinoma, and never-smokers and a lower *EGFR* mutation rate compared with older patients.

## INTRODUCTION

Lung cancer is the leading cause of malignancy-related mortality worldwide [1]. There are fewer studies on young lung cancer patients, i.e., those less than 40 years of age, owing to the fact that the median age of lung cancer at diagnosis is 65–70 years [2]. Among all non-small cell lung cancer (NSCLC) patients, 0.6% ∼ 5.3% is the young lung cancer, defined as less than 40∼45 years [2–5]. Female gender and adenocarcinoma predominance are consistent features, but survival data and late stage at diagnosis yield conflicting findings in young lung cancer in previous reports [2–6]. Furthermore, smoking status and *EGFR* mutation were important factors in the current lung cancer study. Majority of lung cancer patients were advanced or metastatic at diagnosis. In the treatment of advanced NSCLC, the first-line use of gefitinib or erlotinib, an orally administered tyrosine kinase inhibitors (TKIs) of *EGFR*, was recommended for patients harboring *EGFR* mutation with improvement of the progression-free survival and acceptable toxicity [7, 8]. The epidemiological study of *EGFR* mutations demonstrated higher frequency among adenocarcinoma histology, never-smoking status, and Asian ethnicity [9–11]. *Anaplastic lymphoma kinase* (*ALK*) rearrangement was another targetable genotype in NSCLC. Crizotinib, an oral small-molecule tyrosine kinase inhibitor of *ALK*, improved the progression-free survival and quality of life in *ALK*-positive patients [12, 13].

To our knowledge, no large-scale survey of Asian young lung cancer with detailed data on smoking and *EGFR* status has been conducted. Only one retrospective study at a single institution has been previously conducted [14], according to our review of the literature. The main objective of this nationwide study was to investigate the characteristics of young adult lung cancer in Taiwan, especially the relationships among smoking behavior, *EGFR* mutation, and age. Therefore, we analyzed the National Taiwan Cancer Registry database for the period 2011–2012. Detailed smoking status and *EGFR* results have been routinely surveyed and documented in the database since 2011.

## RESULTS

The database included 21,536 patients (13,187 men and 8349 women) diagnosed with lung cancer from 2011 to 2012 in Taiwan. Among these patients, 1074 (5.0%) were in the younger age group (age ≦ 45 years), and 20,462 patients (95.0%) were in the older age group (Table 1). There was a greater proportion of females in the younger age group than in the older age group (48.8% versus 38.2%, *P* < 0.001). The proportion of never-smokers was significantly higher in the younger than in the older group (47.3% versus 43.8%, *P*= 0.015). Adenocarcinomas were more frequent in the younger than in the older group (70.4% versus 58.1%, *P* < 0.001). The primary site of lung cancer was not significantly different between the two groups (56.2% versus 57.7% in upper lobes, *P* = 0.910). The distribution of stage at diagnosis was not significantly different (stage I, 14.9% versus 14.4%; stage II, 4.0% versus 4.2%; stage III 12.9% versus 16.3%; stage IV, 56.6% versus 57.1%, *P* = 0.095 ). Among patients with known smoking status and adenocarcinoma, the *EGFR* mutation test was performed in 59.9% of the younger patients and in 56.1% of the older patients. The *EGFR* mutation rate was significantly lower in the younger patients compared with the older patients (52.5% versus 60.6%, *P* = 0.001).

**Table 1:** Patient characteristics between the younger and older groups in all lung cancer in Taiwan, 2011–2012 (*n* = 21,536)

Demographic characteristics	≦ 45 years old	> 45 years old	*P* value
**Number of cases**	1074	20462	< 0.001
**Gender, *n* (%)**			
Male	550 (51.2)	12637 (61.8)	< 0.001
Female	524 (48.8)	7825 (38.2)	
**Smoking history, *n* (%)**			
Never-smoking	508 (47.3)	8954 (43.8)	0.015
Ever-smoking	419 (39.0)	8702 (42.5)	
NA	147 (13.7)	2806 (13.7)	
**Histology, *n* (%)**			
Adenocarcinoma	756 (70.4)	11892 (58.1)	< 0.001
Squamous cell carcinoma	75 (7.0)	3349 (16.4)	
Small cell carcinoma	43 (4.0)	1647 ( 8.0)	
Other types	200 (18.6)	3574 (17.5)	
**Location, *n* (%)**			
Upper lobes	604 (56.2)	11800 (57.7)	0.910
Lower lobes	341 (31.8)	6714 (32.8)	
NA	129 (12.0)	1948 ( 9.5)	
**Stage, *n* (%)**			
Stage I	160 (14.9)	2943 (14.4)	0.095
Stage II	43 (4.0)	860 ( 4.2)	
Stage III	139 (12.9)	3338 (16.3)	
Stage IV	608 (56.6)	11674 (57.1)	
NA	124 (11.5)	1647 ( 8.0)	
*EGFR* testing*, *n* (%)			
Performed	406 (59.9)	6077 (56.1)	0.129
Not performed	282 (40.1)	4764 (43.9)	
*EGFR* mutation*, *n* (%)			
Positive	213 (52.5)	3684 (60.6)	0.001
Negative	193 (47.5)	2393 (39.4)	

Abbreviations: NA, not available; *EGFR*, epidermal growth factor receptor.

* Only adenocarcinoma patients with known smoking status were included for analysis.

As shown in Table 1, younger patients are more likely to be female, never-smokers, adenocarcinoma, and harbor wild type *EGFR* gene. In our study cohort, 47.3% of the younger patients and 43.8% of the older patients are never-smokers, and smoking behavior might be a complex confounding factor. We selected never-smoking lung cancer for further analysis. As shown in Table 2, the younger age group was significantly more likely to have wild-type *EGFR* (OR = 1.68, 95% CI: 1.30∼2.17, *P* < 0.001), and stage IIIB/IV cancer at diagnosis than the older group (OR = 1.84, 95% CI: 1.22∼2.76, *P* = 0.003).

**Table 2:** Characteristics between younger and older groups for never-smoking lung cancer patients with performed *EGFR* testing (*n *= 4,440)

	≦ 45 years old	> 45 years old	Odds ratio*	*P* value
**Gender, *n* (%)**				
Male	63 (24.0)	1011 (24.2)	1.00 (reference)	
Female	199 (76.0)	3167 (75.8)	1.03 (0.77∼1.39)	0.955
**Histology, *n* (%)**				
Adenocarcinoma	249 (95.0)	3952 (94.6)	1.00 (reference)	
Others	13 ( 5.0)	226 ( 5.4)	0.91 (0.51∼1.62)	0.756
**Stage, *n* (%)**				
I–IIIA	27 (10.3)	729 (17.4)	1.00 (reference)	
IIIB–IV	235 (89.7)	3449 (82.6)	1.84 (1.22∼2.76)	0.003
***EGFR* mutation, *n* (%)**				
Positive	148 (56.5)	2859 (68.4)	1.00 (reference)	
Negative	114 (43.5)	1319 (31.6)	1.68 (1.30∼2.17)	< 0.001

* Odds ratios for younger age group.

As shown in Table 3, we used multiple logistic regression to analyze *EGFR* mutation status in lung adenocarcinoma patients. Older patients (OR: 1.38, 95% CI: 1.12∼1.69, *P* = 0.001) and females (OR: 1.19, 95% CI: 1.04∼1.36, *P* < 0.001) were significantly more likely to have *EGFR* mutation. Ever-smokers were significantly more likely to have *EGFR* wild-type (OR: 0.42, 95% CI: 0.36∼0.48, *P* < 0.001). In above multiple logistic regression, age is an independent factor to predict *EGFR* mutation status.

**Table 3:** Multiple logistic regression for *EGFR* mutation in lung adenocarcinoma patients (*n* = 6483)

	*EGFR* Mutation	*EGFR* Wild-type	Odds ratio*	*P* value
**Gender, *n* (%)**				
Male	1586 (40.7)	1511 (58.4)	1.00 (reference)	
Female	2311 (59.3)	1075 (41.6)	1.19 (1.04–1.36)	< .001
**Age, *n* (%)**				
**≦ 45 years old**	213 ( 5.5)	193 ( 7.5)	1.00 (reference)	
> 45 years old	3684 (94.5)	2393 (92.5)	1.38 (1.12–1.69)	0.001
**Stage, *n* (%)**				
I–IIIA	635 (16.3)	378 (14.6)	1.00 (reference)	
IIIB–IV	3262 (83.7)	2208 (85.4)	0.95 (0.82–1.09)	0.068
**Smoking history, *n* (%)**				
Never smoker	2878 (73.9)	1323 (51.2)	1.00 (reference)	
Ever smoker	1019 (26.1)	1263 (48.4)	0.42 (0.36–0.48)	< .001

Abbreviations: *EGFR*, epidermal growth factor receptor.

* Odds ratios for *EGFR* mutation.

**Figure 1:**
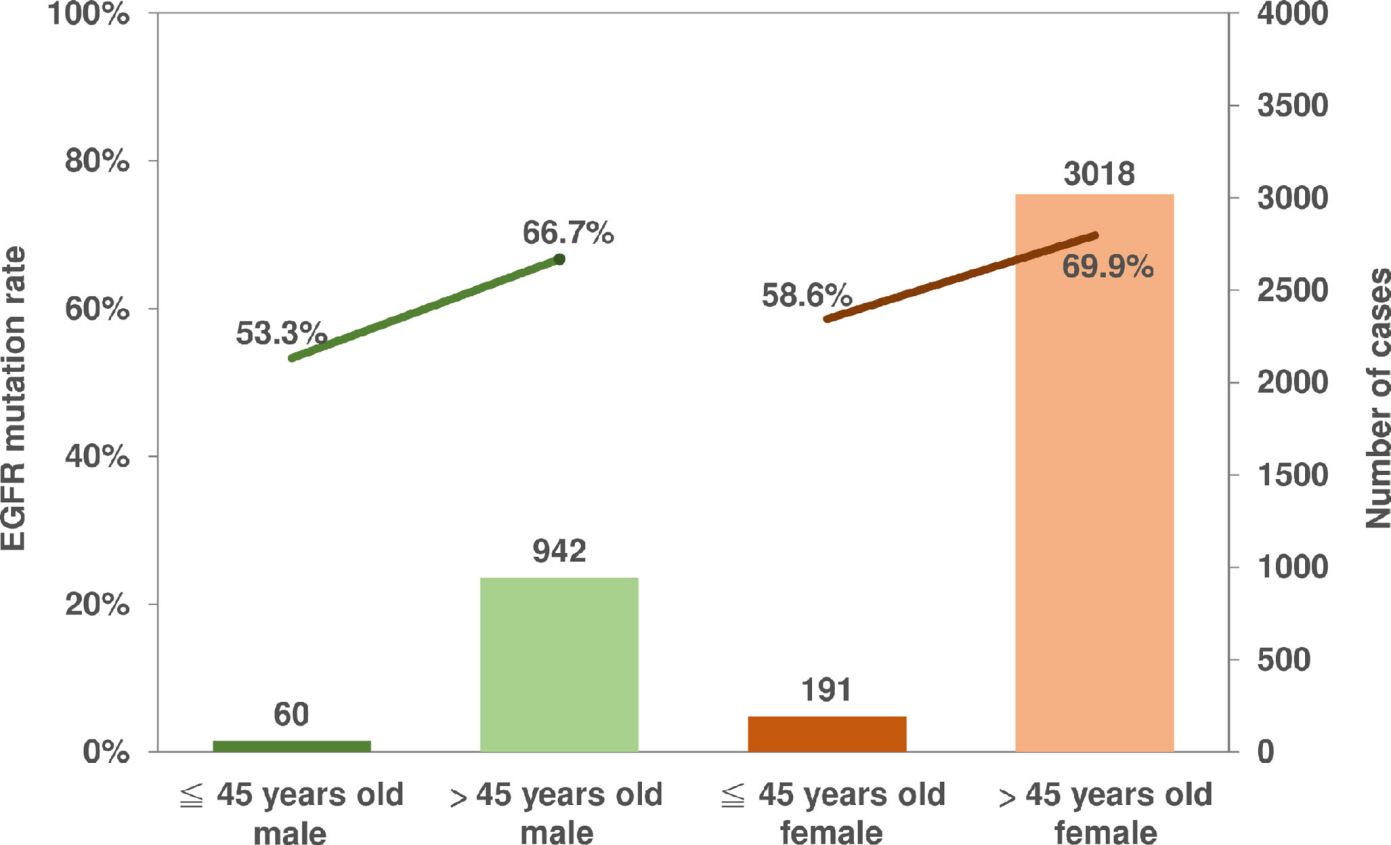
*EGFR* mutation status in never-smoking adenocarcinoma patients with respect to different age groups and genders

Figure 1 shows the *EGFR* mutation status in never-smoking adenocarcinoma patients with respect to different age groups and genders. The *EGFR* mutation rate was significantly lower in the younger age group than in the older age group (53.3% versus 66.7%, *P* = 0.035) in never-smoking male patients with adenocarcinoma; and also in never-smoking female patients with adenocarcinoma (58.6% versus 69.9%, *P* = 0.001).

## DISCUSSION

Lung cancer is more common among older patients. Young lung cancer, which was defined as lung cancer occurring in individuals less than or equal to 45 years of age, accounted for about 5.0% of total lung cancer patients in this study. We analyzed the younger patients based on the National Taiwan Cancer Registry database from 2011 to 2012. The clinical characteristics of younger age patients showed unique factors compared to older patients, such as greater proportions of female patients, adenocarcinoma, and never-smoking, and a lower *EGFR* mutation rate. There were no significant differences in the stage distribution and primary lung cancer location between younger and older patients.

Smoking has a major impact on the risk of developing lung cancer. In our analysis, there was a significant difference in smoking behavior between the two age groups. There were more never-smokers in the younger age group. However, the never-smoking rate even in the older age group was much higher when compared with the rate reported in the French nationwide NSCLC study (43.8% vs. 19.0%) [15]. This is one of the major differences in lung cancer between Asian and Caucasian patients. Furthermore, in our analysis, among lung adenocarcinoma patients with detailed smoking and *EGFR* mutation status, there was an even higher rate of never-smokers (64.8%).

The incidence of young lung cancer in the SEER database showed a decreasing trend during the period 1978 to 2010, but an increasing trend was found in other reports. The phenomenon may be due to the divergent smoking prevalence rates reported in different studies [4, 5, 16, 17]. Moreover, other hypotheses such as genes involved in xenobiotic metabolizing enzymes [18], DNA repair [19], and occupational risk factors have also been reported [20]. Tobacco smoking may result in various types of DNA damage in lung parenchyma and may lead to chronic inflammation, ultimately leading to carcinogenesis. According to the hypothesis of genetic variation in DNA repair and cell cycle control genes, young lung cancer patients may be more susceptible to carcinogens in tobacco smoke than older patients. Landi et al. surveyed 299 lung cancer cases diagnosed before the age of 50 years and 317 age-matched controls patients from six countries in Central and Eastern Europe. They found a significant association with polymorphisms in genes involved in DNA damage sensing and in four genes encoding proteins involved in mismatch repair (*LIG1, LIG3, MLH1*, and *MSH6*). Young patients with specific polymorphisms of the above genes may be more susceptible to carcinogens [19].

Our results are consistent with previous studies that found a greater proportion of female patients and adenocarcinoma in the younger age group, but there were no significant differences in primary lung cancer location and stage at diagnosis between younger and older patients [2, 5]. Moreover, the *EGFR* mutation rate of younger lung cancer patients was significantly lower than that in the older group. To our knowledge, this is the first study to analyze the aforementioned factors using a nationwide database.

*EGFR* mutation was associated with female, adenocarcinoma histology, never-smoking status, and Asian ethnicity [9–11]. Regarding *EGFR* status in our study, there was a lower *EGFR* mutation rate in the younger group. We used *EGFR* mutation as the outcome in a further analysis. Older patient, never-smokers, and females were significantly more likely to have the *EGFR* mutation according to the results of the multiple logistic regression analysis. As there was a significantly higher proportion of never-smokers in the younger patient, why did younger lung adenocarcinoma patients have a lower *EGFR* mutation rate than that of older patients? There are a couple of possible reasons. First, besides *EGFR* mutation, there are several molecular alterations involved in lung adenocarcinoma carcinogenesis, including *v-raf murine sarcoma viral oncogene homolog B* (*BRAF*), *Kirsten rat sarcoma viral oncogene homolog* (*KRAS*), *human epidermal growth factor receptor 2* (*HER2*), and *echinoderm microtubule-associated protein-like 4-anaplastic lymphoma kinase* (*EML4-ALK*). Recent data have suggested that *ALK* rearrangement lung cancers are associated with a younger age at diagnosis [21–23]. In our previous study of the mutation status of five genes in lung adenocarcinoma, *EGFR* mutations were more common in female patients and non-smokers. In *EGFR* wild-type adenocarcinoma, the proportion of *EML4-ALK* translocation was 9.8% and predominant in patients younger than 65 years (14% versus 3.4%) [24]. With further analysis, we found the mean age of *ALK*-positive patients were about 11 years younger than that of *EGFR* mutation patients (Supplementary Table 1). In a study by Guo et al., 95 Chinese never-smoking males were diagnosed with NSCLC. The proportion of *EML4-ALK* translocation was 8.4% (8 out of 95 patients), and was more frequently found in never-smokers and younger patients (5 patients between 40∼49 year-old) [25]. In the recent study of Sacher et al., they reported that the younger age patients are associated with an increased likelihood of harboring a targetable genotype, which *EGFR* mutation (*P* = 0.02) and *ALK* rearrangement (*P* < 0.01) were significantly associated with younger age. In their cohort, those younger patients (less than 40 years old) are more likely to be never smokers (67%), and older patients (more than 60 years old) are less likely to be never smokers (22%). Under multivariable analysis, the association between *EGFR* mutations and age was not significant [26]. ALK rearrangements may explain at least in part why younger patients had a lower positive *EGFR* mutation rate in this study.

Second, in our study, female lung adenocarcinoma patients had a higher *EGFR* mutation rate than that of male patients. We had a greater proportion of female lung cancer patients in the younger group, but the *EGFR* mutation rate was lower than that of the older group. Moreover, with respect to *EML4-ALK* translocation, there may be a different pattern of carcinogenesis between the younger and older groups. The contributing factors may involve genetic susceptibility, environmental factors, and their complex interactions in the pathogenesis of lung cancer in younger patients.

In our study, smoking and *EGFR* mutation status were registered nationwide. This is the first large-scale study on lung cancer to include detailed information pertaining to both smoking and *EGFR* mutation. In future studies of young lung cancer and its association with the *EGFR* mutation, additional factors should be included in the analysis, such as family lung cancer history, smoking index, age of starting smoking, passive smoking, `environmental exposures, and status of other driver oncogene, including *ALK* rearrangement, *BRAF*, *KRAS*, *ROS-1* and *HER-2*.

In conclusion, our findings demonstrated that lung cancer in younger patients (≦ 45 years) has unique characteristics, with a greater proportion of female patients, adenocarcinoma, and never-smokers, and a lower *EGFR* mutation rate compared with older patients. The stage distribution and location of the primary tumor were not significantly different between the two groups.

## MATERIALS AND METHODS

Since 2011, collection of detailed smoking history status was officially included in the National Taiwan Lung Cancer Registry database and *EGFR* mutation exams were routinely surveyed from lung adenocarcinoma patients most in advanced stages for EGFR-TKI treatment evaluation. We analyzed data for the period from 2011 to 2012 in the present study. Lung cancer histology was classified according to the World Health Organization criteria [27]. Demographic characteristics, and clinical data, including age, gender, tumor stage, primary tumor location, smoking status, and *EGFR* mutation were included in the analysis. Never-smokers were defined as patients who had never smoked cigarettes, whereas ever-smokers were defined as those who were current or former smokers. This study was approved by the Institutional Review Board of Taiwan's National Health Research Institutes Research Ethics Committee (IRB No. EC1031002-E)

There are a few different methods for *EGFR* mutation testing in Taiwan, including direct sequencing, and mutant type-specific sensitive methods, such as protein nucleic acid-locked nucleic acid polymerase chain reaction (PNA-LNA PCR) clamp, scorpions amplification refractory mutation system (ARMS) and Cobas *EGFR* Mutation Test. Among all hospitals in Taiwan, there are different laboratory facilities and preferences with respect to *EGFR* mutation testing. The lung cancer stages were all based on the American Joint Committee on Cancer (AJCC) 7th edition. We classified young lung cancer as age below or equal to 45 years. Missing data in the database were also analyzed to determine whether or not they were missing-at-random. Chi-square test was conducted to analyze patients’ characteristics. Multivariable analysis of the correlation of *EGFR* mutation and age with other factors was performed using logistic regression. All analyses were performed using SAS version 9.3 statistical software (SAS Institute, Cary, NC, USA)..

## Supplementary Materials and Table


