# Supplementary material for: Characteristics of young lung cancer: Analysis of Taiwan's nationwide lung cancer registry focusing on epidermal growth factor receptor mutation and smoking status
Source: Oncotarget. 2016 May 13;7(29):46628–35. doi: 10.18632/oncotarget.9338 (PMC5216823; doi:10.18632/oncotarget.9338)
Supplement: Supplementary file 1 [file oncotarget-07-46628-s001.pdf]

Characteristics of young lung cancer: Analysis of Taiwan's nationwide lung cancer registry focusing on epidermal growth factor receptor mutation and smoking status

Supplementary Materials

Supplementary Table S1: Mean age in each genotype group

	EGFR	*ALK*	*KRAS*	*HER-2*
**Positive**	64.1	53.4	65.6	61.0
**Negative**	63.3	64.3	63.7	63.8
